# Topological degeneracy breaking in synthetic frequency lattice by Floquet engineering

**DOI:** 10.1515/nanoph-2023-0408

**Published:** 2023-09-25

**Authors:** Xin Qiao, Luojia Wang, Guangzhen Li, Xianfeng Chen, Luqi Yuan

**Affiliations:** State Key Laboratory of Advanced Optical Communication Systems and Networks, School of Physics and Astronomy, Shanghai Jiao Tong University, Shanghai 200240, China; Collaborative Innovation Center of Light Manipulations and Applications, Shandong Normal University, Jinan 250358, China; Shanghai Research Center for Quantum Sciences, Shanghai 201315, China

**Keywords:** synthetic frequency dimensions, Floquet engineering, topologically edge states

## Abstract

Synthetic frequency dimensions provide important opportunities to investigate novel topological phenomena. Previously, many theoretical proposals have been studied and relevant experiments have been performed very recently. However, all these works consider models in the weak modulation regime, where static lattice models are constructed. Here we explore a Floquet Su–Schrieffer–Heeger (SSH) model with time-dependent hoppings in the frequency dimension by dynamically modulating ring resonators ultrastrongly, and find that the topological states, originally degenerate in conventional SSH lattices, are separated in eigenenergies. There exists a series of edge states from band folding at the 0 and *π* energy bandgaps, which exhibit complex multi-frequency oscillations due to the inclusion of counter-rotating terms with higher order oscillation frequencies. Such a system with stronger modulations can widen the bandgap and therefore it provides an effective way to localize pulses in synthetic frequency dimensions. Our work shows a photonic platform with the synthetic dimension in exploring exotic Floquet topological phenomena and shows potential applications in optical storage and communications.

## 1 Introduction

Floquet engineering provides a powerful tool for exploring physics under periodic drives [1–6] from condensed matter systems [7, 8] to photonic platforms [9–13], giving rise to various schemes for manipulating physical states. In particular, the introduction of temporal modulations with periodicity allows one to explore novel states of matter, such as Floquet topological physics [14–25]. Compared with their static counterparts, the Floquet systems exhibit periodic bandstructure in the time domain, resulting in anomalous topologically protected edge states with zero Chern number [20, 26, 27], which can be manipulated via the external drives and hence lead to active light manipulations [28–31].

Synthetic frequency dimensions, constructed by periodically modulating the refractive index of the ring resonator, have attracted extensive attentions in both theories and experiments [32–36]. Such a system with the external periodic driving supports a tight-binding lattice as the modulation connects discrete resonant frequency modes in the ring, which usually does not require the Floquet analysis in the weak coupling limit with the rotating-wave approximation (RWA) [37, 38]. However, once the modulation is strong enough to break RWA, the resulting synthetic lattice is naturally a Floquet system with non-negligible couplings from counter-rotating terms [39–45], which still remains unexplored in details. In addition, the diverse arrangements of resonators and modulation formats make it easy to create synthetic space with three or even higher dimensions [46–48]. This provides unparalleled advantages in the future efforts in achieving high-dimensional synthetic lattices with the Floquet engineering beyond RWA.

In this paper, we study a Floquet Su–Schrieffer–Heeger (SSH) lattice in the synthetic frequency dimension, which is constructed by dynamically modulating rings ultrastrongly, and find the degeneracy break of topological modes. The conventional SSH model [49] as one of the widely-explored examples in topological physics, has been extensively studied in the field of optics due to its simplicity and intuitive nature [50–54], which shows a pair of degenerate zero-energy modes in the non-trivial topological phase. Here, we consider a realistic theoretical model based on the previous experiment in building the synthetic SSH lattice [55] but under the ultrastrong modulation limit. Such a system shows the existence of a pair of topological modes that hold opposite eigenenergies and can be effectively excited on a particular boundary by selectively choosing the frequency of the excitation light field. In addition, we find there are a series of edge states caused by band folding at the 0 and *π* energy bandgaps that display oscillations resulting from counter-rotating terms with high-order oscillation frequencies. The ultrastrong coupling in the synthetic SSH lattice can further widen the bandgap, which offers an excellent opportunity for localizing energy from the pulse excitation in the frequency dimensions. Our work unveils novel topological phenomena in a synthetic lattice under the Floquet engineering, and may find potential applications in optical storage and communications [56].

## 2 Model

**Figure 1:**
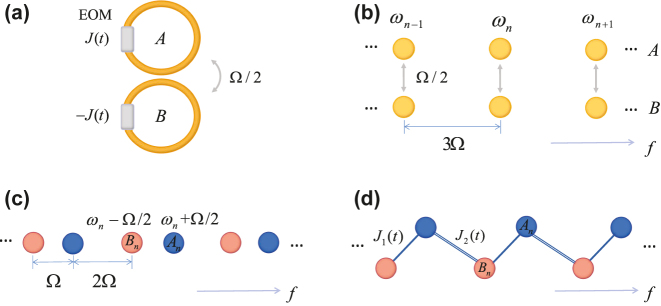
Construction of 1D Floquet SSH model in synthetic frequency dimension. (a) Two identical ring resonators undergo antisymmetric dynamic modulations *J*(*t*) and −*J*(*t*) are coupled at the coupling strength Ω/2. (b) Each ring supports same equally-spaced resonant modes. (c) After the effective coupling between two rings, the antisymmetric and symmetric supermodes (*B*_*n*_ and *A*_*n*_) at the frequency *ω*_*n*_ − Ω/2 and *ω*_*n*_ + Ω/2 are created, which are separated at alternating spacing Ω and 2Ω in the frequency dimension. (d) The Floquet SSH lattice in Eq. (3) in the synthetic frequency dimension.

We consider an SSH model in the synthetic frequency dimension based on the recent experiment [55], which is briefly summarized in the following. A pair of coupled ring resonators with the same length *L* are considered as shown in Figure 1(a). In each ring, the resonant modes are equally spaced along the frequency axis of light with the free spectral range (FSR) Ω_FSR_/2*π* = *ν*_g_/*L*, where *ν*_g_ is the group velocity of light if ignoring the group velocity dispersion. We set Ω_FSR_ = 3Ω for simplicity so the frequency for the *n*th mode reads *ω*_*n*_ = *ω*_0_ + 3*n*Ω, where *ω*_0_ is a reference frequency [see Figure 1(b)]. We further couple two rings at the strength Ω/2, which hybridizes the same resonant modes into photonic molecule [57] with a pair of antisymmetric and symmetric supermodes (*B*_*n*_ and *A*_*n*_) at the frequency *ω*_*n*_ − Ω/2 and *ω*_*n*_ + Ω/2. This creates synthetic sites with alternating spacing Ω and 2Ω in the frequency dimension [see Figure 1(c)], which can be tuned by choosing different coupling strength (see Supplementary Materials Section I). A pair of electro-optic modulators with asymmetric periodic modulations ±*J*(*t*) are added into the ring to modulate the system at frequencies Ω and 2Ω simultaneously, so the synthetic lattice can be built [55] and the corresponding Hamiltonian reads:

(1) $$\begin{array}{ll} H & =\underset{n}{\sum }\left(\omega_{n}+\frac{\Omega }{2}\right)\;a_{n}a_{n}^{†}+\underset{n}{\sum }\left(\omega_{n}-\frac{\Omega }{2}\right)b_{n}b_{n}^{†}\\ & \;+\underset{n}{\sum }J\left(t\right)\left(a_{n}^{†}b_{n}+a_{n-1}b_{n}^{†}+\text{h.c.}\right), \end{array}$$

where

(2) $$J\left(t\right)=2g_{1}\cos\left(\Omega t+\phi_{1}\right)+2g_{2}\cos\left(2\Omega t+\phi_{2}\right).$$

Here 2*g*_1_, 2*g*_2_ are the modulation amplitudes and *ϕ*_1_, *ϕ*_2_ are the modulation phases. By taking ${\tilde{a}}_{n}=a_{n}{\text{e}}^{\text{i}n\left(\omega_{n}+\frac{\Omega }{2}\right)t}$ and ${\tilde{b}}_{n}=b_{n}{\text{e}}^{\text{i}n\left(\omega_{n}-\frac{\Omega }{2}\right)t}$ , we can transform the Hamiltonian (1) to

(3) $$\tilde{H}=\underset{n}{\sum }J_{1}\left(t\right){\tilde{a}}_{n}^{†}{\tilde{b}}_{n}+\underset{n}{\sum }J_{2}\left(t\right){\tilde{a}}_{n-1}{\tilde{b}}_{n}^{†}+\text{ h.c.},$$

where $J_{1}\left(t\right)=g_{1}\left[{\text{e}}^{\text{i}\left(2\Omega t+\phi_{1}\right)}+{\text{e}}^{-\text{i}\phi_{1}}\right]+g_{2}\left[{\text{e}}^{\text{i}\left(3\Omega t+\phi_{2}\right)}\right.\left.+,{\text{e}}^{-\text{i}\left(\Omega t+\phi_{2}\right)}\right]$ and $J_{2}\left(t\right)=g_{1}\left[{\text{e}}^{\text{i}\left(3\Omega t+\phi_{1}\right)}+{\text{e}}^{-\text{i}\left(-\Omega t+\phi_{1}\right)}\right]+g_{2}\left[{\text{e}}^{\text{i}\left(4\Omega t+\phi_{2}\right)}+{\text{e}}^{-\text{i}\phi_{2}}\right]$ . In the weak coupling regime 2*g*_1_, 2*g*_2_ ≪ Ω, the Hamiltonian (3) can be reduced to a conventional SSH model in the recent experiment [55]

(4) $${\tilde{H}}_{\text{RWA}}=\underset{n}{\sum }g_{1}{\text{e}}^{-\text{i}\phi_{1}}{\tilde{a}}_{n}^{†}{\tilde{b}}_{n}+\underset{n}{\sum }g_{2}{\text{e}}^{-\text{i}\phi_{2}}{\tilde{a}}_{n-1}{\tilde{b}}_{n}^{†}+\text{h.c.},$$

under RWA, where *g*_1_ and *g*_2_ indicate the intracell and intercell hopping strengths, respectively. However, for the case under ultrastrong couplings, RWA is broken and all counter-rotating terms in Eq. (3) cannot be simply dropped, so the system gives the synthetic Floquet SSH lattice, which offers opportunities towards exotic topological phenomena.

The system can also be explored by numerically solving the Hamiltonian in the synthetic frequency SSH model. Specifically, the synthetic lattice can be excited by injecting the light through an external waveguide in the vicinity of the single frequency *ω*_*N*,*a*_ = *ω*_*N*_ + Ω/2 (*ω*_−*N*,*b*_ = *ω*_−*N*_ − Ω/2), i.e., to excite the single mode *A*_6_ (*B*_−6_) at the artificial boundary. Output signals are also collected by the same external waveguide. Therefore, the coupled-mode equations for describing the Hamiltonian (3) with the input–output channels are [58, 59]

(5) $$\begin{array}{cc} {\dot{a}}_{n} & =-\text{i}J\left(t\right)\left(b_{n}{\text{e}}^{\text{i}\Omega t}+b_{n+1}{\text{e}}^{-\text{i}2\Omega t}\right)+\text{i}\sqrt{\gamma }s_{a,n}^{\text{in}},\\ {\dot{b}}_{n} & =-\text{i}J\left(t\right)\left(a_{n}{\text{e}}^{-\text{i}\Omega t}+a_{n-1}{\text{e}}^{\text{i}2\Omega t}\right)+\text{i}\sqrt{\gamma }s_{b,n}^{\text{in}}, \end{array}$$

(6) $$\begin{array}{cc} s_{a,n}^{\text{out}} & =s_{a,n}^{\text{in}}+\text{i}\sqrt{\gamma }a_{n},\\ s_{b,n}^{\text{out}} & =s_{b,n}^{\text{in}}+\text{i}\sqrt{\gamma }b_{n}, \end{array}$$

where *γ* is the coupling strength between waveguides and rings, which is taken as *γ* = 0.01Ω throughout simulations, $s_{a,n}^{\text{in}}$ and $s_{b,n}^{\text{in}}$ ( $s_{a,n}^{\text{out}}$ and $s_{b,n}^{\text{out}}$ ) are the input (output) amplitudes of the optical field, respectively. Here $s_{a,n}^{\text{in}}=\delta_{n,N}{\text{e}}^{-\text{i}\Delta \varepsilon t}$ and $s_{b,n}^{\text{in}}=\delta_{n,-N}{\text{e}}^{-\text{i}\Delta \varepsilon t}$ , where Δ*ε* is the small frequency offset in the single-frequency input field to excite a particular band.

## 3 Floquet quasienergy bandstructure and edge states

To explore the topological properties from the synthetic SSH model beyond RWA in Eq. (3), we apply the Floquet analysis [1, 3, 42, 60]. The Hamiltonian (3) holds periodicity in time as *T* = 2*π*/Ω so it supports $\tilde{H}\left(t+T\right)=\tilde{H}\left(t\right)$ . One can then take a quasi-stationary Floquet state $\left|\psi \right\rangle ={\text{e}}^{-\text{i}\varepsilon t/\hbar }\left|\Phi \right\rangle$ , where $\left|\Phi \left(t+T\right)\right\rangle =\left|\Phi \left(t\right)\right\rangle$ is the Floquet eigenstate, and *ε* is the Floquet quasienergy with the temporal first Brillouin zone −Ω/2 ≤ *ε* ≤ Ω/2 and use the Floquet ansatz for eigenvalues of the Schrödinger equation, which gives $\tilde{H}-\text{i}\frac{\partial }{\partial t}|\Phi 〉=\varepsilon |\Phi 〉$ . Such an equation can be expressed as an eigenvalue problem in the Fourier harmonic space. The Floquet quasienergy spectrum can then be obtained by diagonalizing the matrix of this extended space with a proper truncation [1, 3, 42].

**Figure 2:**
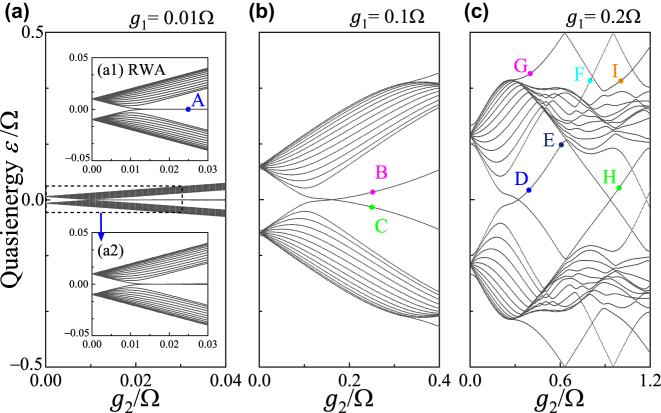
Floquet quasienergy bandstructure of the synthetic frequency SSH model as a function of modulation amplitudes *g*_2_ with (a) *g*_1_ = 0.01Ω, (b) *g*_1_ = 0.1Ω and (c) *g*_1_ = 0.2Ω. Comparison of the energy bandstructure with (a1) RWA from ${\tilde{H}}_{\text{RWA}}$ and (a2) the Floquet case in the weak coupling regime. Eigenvalues labelled by A–I will be analyzed in simulations later.

To examine the topological edge states in such SSH lattice beyond RWA, we consider the open-boundary case, which is technically achievable by creating the artificial boundary along the synthetic frequency dimension [61]. Specifically, we consider 13 resonant modes (*n* = −6, …, 6), which gives 13 pairs of supermodes (*A*_*n*_ and *B*_*n*_). *ϕ*_1_ = *ϕ*_2_ = 0 is taken for the simplicity. We plot Floquet quasienergy bandstructure versus *g*_2_ in Figure 2, with different amplitudes of *g*_1_ ranging from weak to ultrastrong couplings, in the first Floquet Brillouin zone as the Floquet bandstructure is periodic for each Ω. In particular, the bandstructure from *H*_RWA_ under RWA is also plotted in Figure 2(a1) for the comparison. In the weak coupling regime, the bandstructure of the ${\tilde{H}}_{\text{RWA}}$ [see Figure 2(a1)] agrees well with the Floquet bandstructure [see Figure 2(a2)], which confirms the validity of the RWA. It is well-known that for the ordinary SSH model, the system supports a pair of degenerate edge states at *ε* = 0 (zero modes) under the topologically non-trivial case with *g*_1_ < *g*_2_. We find that, if the synthetic SSH model goes beyond RWA at *g*_1_ = 0.1Ω, the degeneracy of zero modes break and varying from positive (negative) energy to negative (positive) energy, respectively, when one increases *g*_2_ larger than *g*_1_ [see Figure 2(b)]. Once *g*_1_ is further increased to 0.2Ω as shown in Figure 2(c), such pair of states get into the bulk states when *g*_2_ $\left(>g_{1}\right)$ is increasing, and in addition, there are other pair of states out of bulk states in-between bulk bandgaps in the vicinity of *ε* ∼ 0 and also for *ε* ≳ (≲) ± 0.5Ω. These additional pairs of anomalous states in-between bulk bandgaps appear from band folding at the 0 and *π* energy bandgaps, which are a unique consequence from the combination of the frequency dimension and the Floquet drive frequency, holding fundamental difference from the edge states of the Floquet SSH lattice model in the real space (see Supplementary Materials Section II).

**Figure 3:**
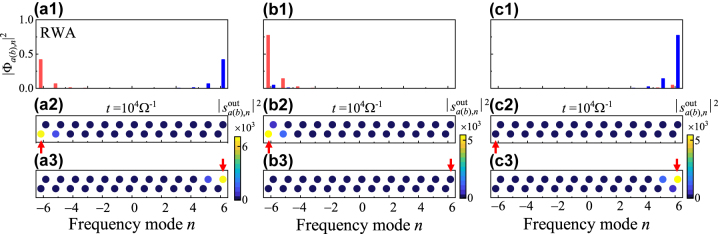
Comparison of eigenstates and simulation results for the localized edge states of the ordinary and Floquet SSH lattice. (a1) Intensity distributions of the eigenstates |Φ_*a*(*b*),*n*_|^2^ for the localized degenerate edge states labelled by A (blue) in Figure 2(a1) with parameters *g*_2_ = 0.025Ω and Δ*ε* = 0. (b1) and (c1) Intensity distributions of the eigenstates |Φ_*a*(*b*),*n*_|^2^ for the localized edge states at B (pink) and C (green) labeled in Figure 2(b) with parameters *g*_2_ = 0.25Ω and Δ*ε* = ±0.0218Ω, respectively. The blue and red bars in (a1)–(c1) indicate sites of supermodes *A*_*n*_ and *B*_*n*_, respectively. (a2)–(c3) Corresponding simulation results of intensity distributions $|s_{a\left(b\right),n}^{\text{out}}{|}^{2}$ for the output field with (a2)–(c2) left boundary excitation and (a3)–(c3) right boundary excitation at the time *t* = 10^4^Ω^−1^. The red arrow indicates the excitation position of the frequency supermode. The upper (lower) column indicates supermodes *A*_*n*_ and *B*_*n*_, respectively.

We next see some typical examples of eigenstate distributions of edge states, together with corresponding simulation results. As shown in Figure 3(a1), we first consider the ordinary SSH model and plot the intensity distributions, |Φ_*a*,*n*_|^2^ and |Φ_*b*,*n*_|^2^, of the degenerate edge states of the ordinary SSH model [see A in Figure 2(a1)]. One can see that such degenerate edge states are localized at boundaries of the lattice and the left (right) edge state has non-zero components only on the frequency supermode *B*_*n*_ (*A*_*n*_), which is well studied [62]. Simulations are performed by exciting the lattice at the left and right boundary, respectively, and the resulting intensity distributions $|s_{a\left(b\right),n}^{\text{out}}{|}^{2}$ for the output field are plotted in Figure 3(a2) and (a3) at the time *t* = 10^4^Ω^−1^, which verifies the exponential decay into the bulk from edge states. Nevertheless, the striking feature of the synthetic Floquet SSH lattice in the ultrastrong coupling regime (*g*_1_ = 0.1Ω) is the breaking of the degeneracy of zero modes, indicated in Figure 2(b). One can see from Figure 3(b1) and (c1) that the intensity distributions of the pair of edge states at *ε* = ±0.0218Ω when *g*_2_ = 0.25Ω exhibit the localization on only one boundary, i.e., the right and left boundaries, respectively. Such features are also verified in numerical simulations [see Figure 3(b2)–(c3)]. In particular, since the left (right) edge state has a positive (negative) eigenvalue, the input field at Δ*ε* = +0.0218Ω (−0.0218Ω) can only excite one left (right) boundary of the synthetic Floquet SSH lattice.

**Figure 4:**
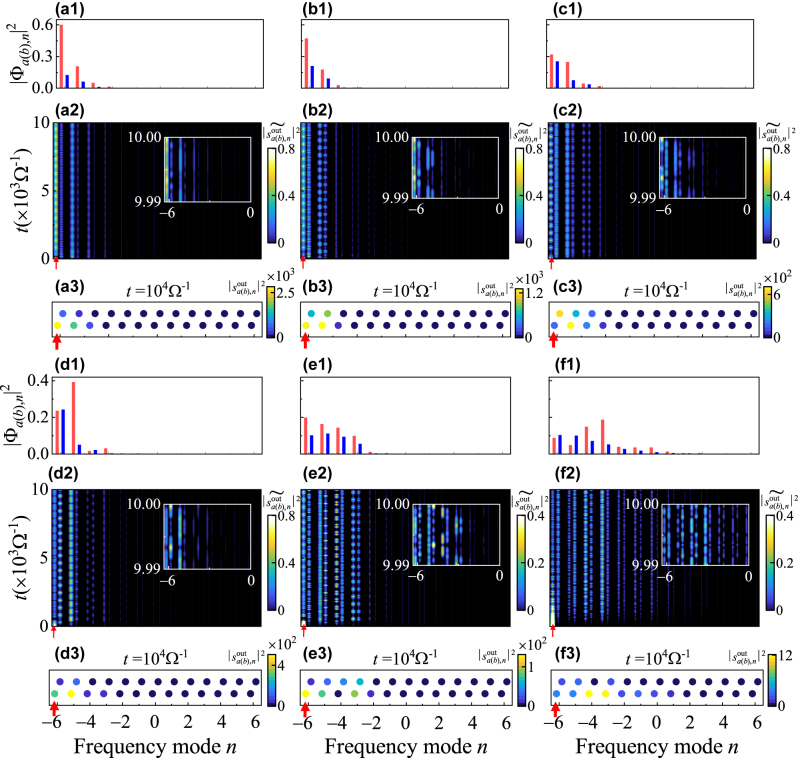
Eigenstates and simulation results for localized edge states from band folding at larger *g*_1_. (a1)–(f1) Intensity projections of the eigenstates |Φ_*a*(*b*),*n*_|^2^ for the localized edge states at (a1) D (blue) labelled in Figure 2(c) with *g*_2_ = 0.4Ω, *ε* = 0.0323Ω; (b1) E (dark blue) with *g*_2_ = 0.6Ω, *ε* = 0.159Ω; (c1) F (light blue) with *g*_2_ = 0.8Ω, *ε* = 0.3553Ω; (d1) G (pink) with *g*_2_ = 0.4Ω, *ε* = 0.3771Ω; (e1) H (green) with *g*_2_ = 1Ω, *ε* = 0.0402Ω and (f1) I (orange) with *g*_2_ = 1Ω, *ε* = 0.3538Ω. (a2)–(f2) The corresponding dynamic evolution of the normalized intensity distributions $|\tilde{s_{a\left(b\right),n}^{\text{out}}}{|}^{2}$ versus the propagation time *t*. Insets show zoom-in evolutions over a short time window Δ*t* = 10Ω^−1^. (a3)–(f3) Corresponding simulation results of intensity distributions $|s_{a\left(b\right),n}^{\text{out}}{|}^{2}$ for the output field at the time *t* = 10^4^Ω^−1^. The red arrow indicates the frequency supermode *B*_−6_ in the left boundary is excited.

We note that, from Figure 2(c), due to the band folding effect once *g*_1_ is further increased to 0.2Ω, additional pairs of states appear at both the 0 and *π* energy bandgaps. We select representative eigenvalues (D to I) on these states at different *g*_2_ and plot the intensity distribution of the corresponding edge states in Figure 4. For the state near *ε* = 0 (D with *g*_2_ = 0.4Ω), the intensity distribution exhibits the localization on the left boundary of the synthetic lattice. This specific state has similar feature as the state shown in Figure 2(b) (marked as B) and Figure 3(b1) for *g*_1_ = 0.1Ω. Nevertheless, we can see that for the larger pair of *g*_1_ and *g*_2_, the intensity of the edge state leaks more into the bulk. We verify the eigenstate distribution with the simulation applied by exciting the synthetic lattice at the left boundary with a corresponding excitation frequency offset Δ*ε* = +0.0323Ω. The energy of the field is largely localized on the left boundary in the simulation. Intriguingly, different from the ordinary SSH zero modes, the edge states studied here with breaking of the degeneracy evolution at the lattice boundary and exhibit complex multi-frequency oscillations as one can see from the normalized intensity distributions $|\tilde{s_{a\left(b\right),n}^{\text{out}}}{|}^{2}$ [in Figure 4(a2)], which results from counter-rotating terms in Eq. (3) carrying high-order oscillation frequencies. When *g*_2_ is increasing, the edge state (the one on which D is) further extends towards the upper bulk bands and then is out of the top of bulk bands. We choose two eigenvalues (E and F) just below and above bulk bands and plot the eigenstate distributions in Figure 4(b1) and (c1). One can see the results also show this band is the edge state where the intensity is mainly focused on the left boundary. Nevertheless, for larger *g*_2_, the distribution shows more energy penetrating into the bulk. Such features are also confirmed by numerical simulations in Figure 4(b2)–(c3). Moreover, from Figure 4(a3)–(c3), we also notice that the total intensities in the synthetic lattice from excitations at the same energy become smaller with the increase of *g*_2_, i.e., the excitation efficiency is decreasing. In Figure 2(c), we can see at *g*_2_ = 0.4Ω, besides the edge state near *ε* = 0, there exists another state, which is actually an edge state at *π*-mode (labelled by G). We also plot its eigenstate intensity distribution in Figure 4(d1), and see the localization effect, verified by the simulation in Figure 4(d2) and (d3). One notes that the normalized intensity distributions $|\tilde{s_{a\left(b\right),n}^{\text{out}}}{|}^{2}$ also exhibit periodic oscillations at *π*-mode, but the total intensity for the output field at the time *t* = 10^4^Ω^−1^ is smaller than the total intensity of the edge state at zero modes with the same *g*_2_ (D). Moreover, for the states generated by multiple foldings, such as H and I at *g*_2_ = 1Ω, we plot the resulting intensity distributions and corresponding simulation results in Figure 4(e1)–(f3). One can see that, although these states still provide some localizations on the left boundary of the synthetic lattice, the effect of localizations and the excitation efficiency are weakened and more energy of the states is distributed into the bulk. These intriguing Floquet topological phenomena are unique in this platform and may originate from the frequency recombination process triggered by the overlapping between the resonant frequency modes and periodic modulations but with strong amplitudes, which can provide new opportunity in manipulating the light in the synthetic frequency dimension. In addition, the complex multi-frequency oscillations in the dynamic evolution of topological modes in the synthetic frequency dimension may have potential applications in the multi-channel information transmission. Further combining the ultrastrong coupling in the synthetic lattice with the nonlinearity can provide a platform to explore physics [63] with the potential emergence of novel optical phenomena and behaviors.

## 4 Localization of pulses in frequency dimension

**Figure 5:**
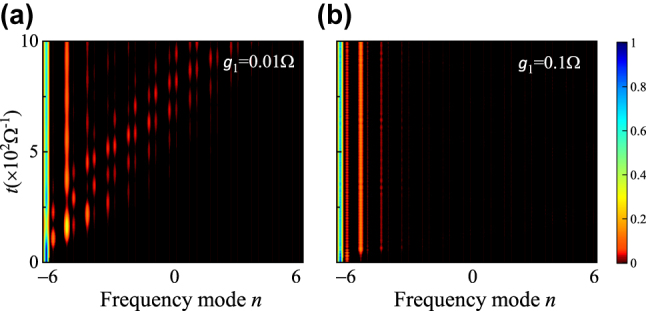
Simulation results of pulse excitations in the synthetic frequency SSH model. (a) The weak coupling regime with *g*_1_ = 0.01Ω, *g*_2_ = 0.025Ω, and Δ*ε* = 0. (b) The ultrastrong coupling regime with *g*_1_ = 0.1Ω, *g*_2_ = 0.25Ω, and Δ*ε* = 0.0218Ω. In both cases, a Gaussian-shape pulse centered at *B*_−6_ supermode is used to excite the lattice.

In the conventional SSH model constructed in the synthetic frequency dimension in the weak coupling limit, although the presence of edge states gives the localized states with resistance to perturbations, the narrow bandgap of the system limits the information carrying capacity of the pulse signals, restricting the development toward practical applications. Nevertheless, the Floquet SSH lattice in the ultrastrong coupling limit greatly increases the size of the bandgap of the system. As demonstrations, we perform simulations using a Gaussian-shape pulse centered at the supermode *B*_−6_ as the excitation source, which reads as $s_{b,n}^{\text{in}}\left(t\right)=\delta_{n,-6}{\text{e}}^{-4\ln2\frac{{\left(\Omega t-50\right)}^{2}}{400}-\text{i}\Delta \varepsilon t}$ . This pulse source carries a temporal full width at half maximum (FWHM) 20Ω^−1^, or a spectral FWHM 0.278Ω. In the system with a fixed Ω, if the system is undergoing modulations in the weak coupling limit, for example, *g*_1_ = 0.01Ω and *g*_2_ = 0.025Ω, the spectral FWHM of the source is much larger than the bandgap. In this case, the topological zero mode [A in Figure 2(a1)] cannot be well excited. We plot the simulation result in Figure 5(a), where one can see that the excitation leaks into the bulk largely. However, if we choose *g*_1_ = 0.1Ω and *g*_2_ = 0.25Ω and excite the non-degenerate edge state [B in Figure 2(b)], we can see the localization feature in Figure 5(b). This type of pulse localization will significantly increase the capacity for carrying information, and may have potential applications in optical storage and communication [64].

## 5 Summary

Our proposal may be realized in a potential experiment based on the established optical fiber setup [55]. Two ring resonators can be coupled using a 2 × 2 fiber coupler with a 50:50 coupling ratio. To achieve ultrastrong coupling, we need to increase the length of the fiber to decreasing omega and also enhance the modulation amplitudes accordingly to break RWA. Furthermore, one can resonantly couple the setup with an additional small auxiliary ring to knock-off a specific supermode in order to induce the sharp boundary in the frequency dimension [61], providing possibilities for observing our theoretical predictions. Recently, relevant experiments have been extended to thin-film lithium niobite-integrated photonic platforms [65, 66], which provides further possible experimental platform in conducting experiments with our proposal.

In summary, we study two coupled ring resonators under the strong modulations that break the RWA and constructs a 1D Floquet SSH lattice in the synthetic frequency dimension. Such a system supports a unique Floquet topological bandstructure, where the originally degenerate topological states are lifted. In addition, a series of edge states caused by band folding at the 0 and *π* energy bandgaps can be found in the ultrastrong coupling regime. Simulations are performed to show the possibilities of exciting particular edge states by selecting the frequency shift of the excitation source. Our results show that the system allows better localization effects of pulses in synthetic frequency dimensions, as the bandgap is enlarged in the ultrastrong coupling limit. The model studied here can be further generalized by adding long-range couplings from where the bandstructure gets further modified compared with that in a conventional SSH lattice (see Section III in Supplementary Materials). Our work hence effectively brings the concept of synthetic frequency dimension into the Floquet problem, and provides a new way towards Floquet topological insulators [14–25], which opens up an exciting avenue for investigating novel Floquet topological phenomena with additional degrees of freedom.

## Supplementary Material

Supplementary Material Details

Supplementary Material Details

## References

[1] Shirley J. H. (1965). Solution of the schrödinger equation with a Hamiltonian periodic in time. *Phys. Rev.*.

[2] Kapitza P. L. (1965). *Collected Papers of PL Kapitza*.

[3] Sambe H. (1973). Steady states and quasienergies of a quantum-mechanical system in an oscillating field. *Phys. Rev. A*.

[4] Wimberger S., Guarneri I., Fishman S. (2004). Classical scaling theory of quantum resonances. *Phys. Rev. Lett.*.

[5] Bukov M., D’Alessio L., Polkovnikov A. (2015). Universal high-frequency behavior of periodically driven systems: from dynamical stabilization to Floquet engineering. *Adv. Phys.*.

[6] Eckardt A. (2017). Colloquium: atomic quantum gases in periodically driven optical lattices. *Rev. Mod. Phys.*.

[7] Oka T., Kitamura S. (2019). Floquet engineering of quantum materials. *Annu. Rev. Condens. Matter Phys.*.

[8] Harper F., Roy R., Rudner M. S., Sondhi S. (2020). Topology and broken symmetry in floquet systems. *Annu. Rev. Condens. Matter Phys.*.

[9] Clark L. W., Schine N., Baum C., Jia N., Simon J. (2020). Observation of Laughlin states made of light. *Nature*.

[10] Park J., Cho H., Lee S. (2022). Revealing non-Hermitian band structure of photonic Floquet media. *Sci. Adv.*.

[11] Jin J., He L., Lu J., Mele E. J., Zhen B. (2022). Floquet quadrupole photonic crystals protected by space-time symmetry. *Phys. Rev. Lett.*.

[12] Yin S., Galiffi E., Alù A. (2022). Floquet metamaterials. *eLight*.

[13] Yuan L., Fan S. (2022). Temporal modulation brings metamaterials into new era. *Light: Sci. Appl.*.

[14] Oka T., Aoki H. (2009). Photovoltaic Hall effect in graphene. *Phys. Rev. B*.

[15] Wang Y., Steinberg H., Jarillo-Herrero P., Gedik N. (2013). Observation of floquet-bloch states on the surface of a topological insulator. *Science*.

[16] Rechtsman M. C., Zeuner J. M., Plotnik Y. (2013). Photonic Floquet topological insulators. *Nature*.

[17] Pasek M., Chong Y. (2014). Network models of photonic Floquet topological insulators. *Phys. Rev. B*.

[18] Zhang Y., Wu Z., Belić M. R. (2015). Photonic Floquet topological insulators in atomic ensembles: photonic Floquet topological insulators in atomic ensembles. *Laser Photonics Rev.*.

[19] Leykam D., Rechtsman M., Chong Y. (2016). Anomalous topological phases and unpaired Dirac cones in photonic floquet topological insulators. *Phys. Rev. Lett.*.

[20] Mukherjee S., Spracklen A., Valiente M. (2017). Experimental observation of anomalous topological edge modes in a slowly driven photonic lattice. *Nat. Commun.*.

[21] Jörg C., Letscher F., Fleischhauer M., von Freymann G. (2017). Dynamic defects in photonic Floquet topological insulators. *New J. Phys.*.

[22] Yang Z., Lustig E., Lumer Y., Segev M. (2020). Photonic Floquet topological insulators in a fractal lattice. *Light: Sci. Appl.*.

[23] Rudner M. S., Lindner N. H. (2020). Band structure engineering and non-equilibrium dynamics in Floquet topological insulators. *Nat. Rev. Phys.*.

[24] Song W., Chen Y., Li H. (2021). Gauge-induced Floquet topological states in photonic waveguides. *Laser Photonics Rev.*.

[25] Nagulu A., Ni X., Kord A. (2022). Chip-scale Floquet topological insulators for 5G wireless systems. *Nat. Electron.*.

[26] Rudner M. S., Lindner N. H., Berg E., Levin M. (2013). Anomalous edge states and the bulk-edge correspondence for periodically driven two-dimensional systems. *Phys. Rev. X*.

[27] Cheng Q., Pan Y., Wang H. (2019). Observation of anomalous π modes in photonic floquet engineering. *Phys. Rev. Lett.*.

[28] Lindner N. H., Refael G., Galitski V. (2011). Floquet topological insulator in semiconductor quantum wells. *Nat. Phys.*.

[29] Cayssol J., Dóra B., Simon F., Moessner R. (2013). Floquet topological insulators. *Phys. Status Solidi RRL*.

[30] Wu S., Song W., Gao S., Chen Y., Zhu S., Li T. (2021). Floquet π mode engineering in non-Hermitian waveguide lattices. *Phys. Rev. Res.*.

[31] Zhong H., Kartashov Y. V., Li Y. (2023). π-mode solitons in photonic Floquet lattices. *Phys. Rev. A*.

[32] Yuan L., Shi Y., Fan S. (2016). Photonic gauge potential in a system with a synthetic frequency dimension. *Opt. Lett.*.

[33] Dutt A., Lin Q., Yuan L., Minkov M., Xiao M., Fan S. (2020). A single photonic cavity with two independent physical synthetic dimensions. *Science*.

[34] Li G., Wang L., Ye R. (2022). Observation of flat-band and band transition in the synthetic space. *Adv. Photonics*.

[35] Cheng D., Wang K., Fan S. (2023). Artificial non-abelian lattice gauge fields for photons in the synthetic frequency dimension. *Phys. Rev. Lett.*.

[36] Yu D., Li G., Wang L., Leykam D., Yuan L., Chen X. (2023). Moiré lattice in one-dimensional synthetic frequency dimension. *Phys. Rev. Lett.*.

[37] Yuan L., Lin Q., Xiao M., Fan S. (2018). Synthetic dimension in photonics. *Optica*.

[38] Yuan L., Dutt A., Fan S. (2021). Synthetic frequency dimensions in dynamically modulated ring resonators. *APL Photonics*.

[39] Günter G., Anappara A. A., Hees J. (2009). Sub-cycle switch-on of ultrastrong light–matter interaction. *Nature*.

[40] Yang S., Al-Amri M., Zhu S.-Y., Zubairy M. S. (2013). Effect of counter-rotating terms on the spontaneous emission in an anisotropic photonic crystal. *Phys. Rev. A*.

[41] Sánchez-Burillo E., Zueco D., Garcia-Ripoll J., Martin-Moreno L. (2014). Scattering in the ultrastrong regime: nonlinear optics with one photon. *Phys. Rev. Lett.*.

[42] Yuan L., Fan S. (2015). Topologically nontrivial Floquet band structure in a system undergoing photonic transitions in the ultrastrong-coupling regime. *Phys. Rev. A*.

[43] Forn-Díaz P., Lamata L., Rico E., Kono J., Solano E. (2019). Ultrastrong coupling regimes of light-matter interaction. *Rev. Mod. Phys.*.

[44] Calvo J., Zueco D., Martin-Moreno L. (2020). Ultrastrong coupling effects in molecular cavity QED. *Nanophotonics*.

[45] Vanhoecke M., Scarlatella O., Schirò M. (2022). ..

[46] Ozawa T., Price H. M., Goldman N., Zilberberg O., Carusotto I. (2016). Synthetic dimensions in integrated photonics: from optical isolation to four-dimensional quantum Hall physics. *Phys. Rev. A*.

[47] Lin Q., Sun X.-Q., Xiao M., Zhang S.-C., Fan S. (2018). A three-dimensional photonic topological insulator using a two-dimensional ring resonator lattice with a synthetic frequency dimension. *Sci. Adv.*.

[48] Yuan L., Xiao M., Lin Q., Fan S. (2018). Synthetic space with arbitrary dimensions in a few rings undergoing dynamic modulation. *Phys. Rev. B*.

[49] Su W., Schrieffer J., Heeger A. J. (1979). Solitons in polyacetylene. *Phys. Rev. Lett.*.

[50] Malkova N., Hromada I., Wang X., Bryant G., Chen Z. (2009). Observation of optical Shockley-like surface states in photonic superlattices. *Opt. Lett.*.

[51] St-Jean P., Goblot V., Galopin E. (2017). Lasing in topological edge states of a one-dimensional lattice. *Nat. Photonics*.

[52] Liu F., Deng H.-Y., Wakabayashi K. (2018). Topological photonic crystals with zero Berry curvature. *Phys. Rev. B*.

[53] Zhu X., Wang H., Gupta S. K. (2020). Photonic non-Hermitian skin effect and non-Bloch bulk-boundary correspondence. *Phys. Rev. Res.*.

[54] Leefmans C., Dutt A., Williams J. (2022). Topological dissipation in a time-multiplexed photonic resonator network. *Nat. Phys.*.

[55] Li G., Wang L., Ye R. (2023). Direct extraction of topological Zak phase with the synthetic dimension. *Light: Sci. Appl.*.

[56] Chen Z., Segev M. (2021). Highlighting photonics: looking into the next decade. *eLight*.

[57] Zhang M., Wang C., Hu Y. (2019). Electronically programmable photonic molecule. *Nat. Photonics*.

[58] Fan S., Suh W., Joannopoulos J. D. (2003). Temporal coupled-mode theory for the Fano resonance in optical resonators. *J. Opt. Soc. Am. A*.

[59] Minkov M., Shi Y., Fan S. (2017). Exact solution to the steady-state dynamics of a periodically modulated resonator. *APL Photonics*.

[60] Rudner M. S., Lindner N. H. (2020). ..

[61] Dutt A., Yuan L., Yang K. Y. (2022). Creating boundaries along a synthetic frequency dimension. *Nat. Commun.*.

[62] Asbóth J. K., Oroszlány L., Pályi A. (2016). A short course on topological insulators. *Lect. Notes Phys.*.

[63] Englebert N., Goldman N., Erkintalo M. (2023). Bloch oscillations of coherently driven dissipative solitons in a synthetic dimension. *Nat. Phys.*.

[64] Amiri I. S., Ahmad H. (2015). *Optical Soliton Communication Using Ultra-Short Pulses*.

[65] Hu Y., Reimer C., Shams-Ansari A., Zhang M., Loncar M. (2020). Realization of high-dimensional frequency crystals in electro-optic microcombs. *Optica*.

[66] Balčytis A., Dinh X. H., Ozawa T. (2023). ..

