# Supplementary material for: Topological degeneracy breaking in synthetic frequency lattice by Floquet engineering
Source: Nanophotonics. 2023 Sep 25;12(19):3807–15. doi: 10.1515/nanoph-2023-0408 (PMC11636413; doi:10.1515/nanoph-2023-0408)
Supplement: Supplementary file 1 — Supplementary Material Details [file j_nanoph-2023-0408_suppl_001.pdf]

Supplementary Material for: Topological degeneracy breaking in synthetic frequency lattice by Floquet engineering

Xin Qiao[1], Luojia Wang[1,*], Guangzhen Li[1], Xianfeng Chen[1,2,3], and Luqi Yuan[1,*]
[1]*State Key Laboratory of Advanced Optical Communication Systems and Networks, School of Physics and Astronomy, Shanghai Jiao Tong University, Shanghai 200240, China*
[2]*Collaborative Innovation Center of Light Manipulations and Applications, Shandong Normal University, Jinan 250358, China and*
[3]*Shanghai Research Center for Quantum Sciences, Shanghai 201315, China*

I. INFLUENCE OF SPACING BETWEEN SUPERMODES

In the main text, the spacing between supermodes is 1:2, i.e., the intracell is Ω and the intercell is 2Ω, which can be tuned by choosing different coupling strength. Here, we set $\Omega_{\mathrm{FSR}} = 4\Omega$, then the antisymmetric dynamic modulations have the form as

$$J(t) = 2g_1 \cos(\Omega t + \phi_1) + 2g_2 \cos(3\Omega t + \phi_2)\,. \tag{S1}$$

Thus, the spacing between supermodes can be changed to 1:3 in the frequency dimension. When transforming Eq. (1) in the main text with $\widetilde{a}_n = a_n e^{in\left(\omega_n + \frac{\Omega}{2}\right)t}$ and $\widetilde{b}_n = b_n e^{in\left(\omega_n - \frac{\Omega}{2}\right)t}$, the Hamiltonian is

$$\begin{aligned}\tilde{H} =& \sum_n \left\{ g_1 \left[e^{i(2\Omega t+\phi_1)} + e^{-i\phi_1} \right] + g_2 \left[e^{i(4\Omega t+\phi_2)} + e^{-i(2\Omega t+\phi_2)} \right] \right\} \tilde{a}_n^\dagger \tilde{b}_n \\ &+ \sum_n \left\{ g_1 \left[e^{i(4\Omega t+\phi_1)} + e^{-i(-2\Omega t+\phi_1)} \right] + g_2 \left[e^{i(6\Omega t+\phi_2)} + e^{-i\phi_2} \right] \right\} \tilde{a}_{n-1} \tilde{b}_n^\dagger + \text{ h.c..}\end{aligned} \tag{S2}$$

By employing the Floquet analysis, we show the quasienergy bandstructure, eigenstate distributions of edge states $|\Phi_{a(b),n}|^2$ and corresponding simulation results of intensity distributions $|s^{\mathrm{out}}_{a(b),n}|^2$ in Fig. S1. As an important subtlety,

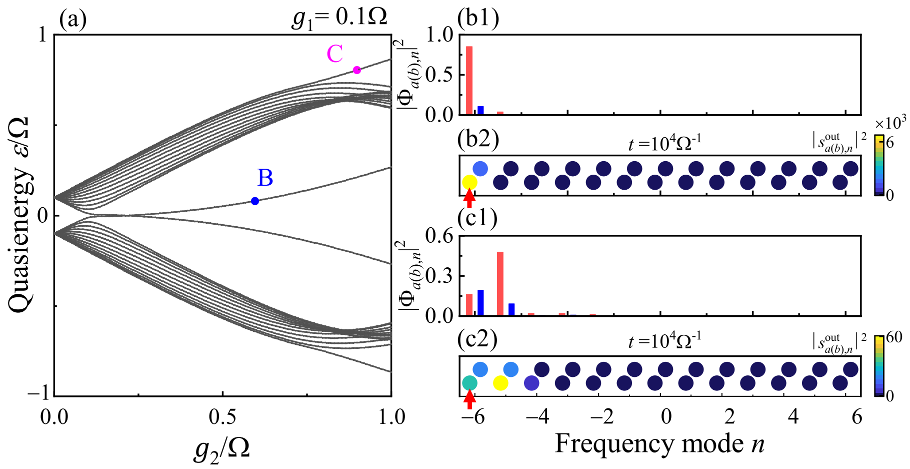

FIG. S1: (color online) (a) Floquet quasienergy bandstructure of the synthetic frequency SSH model as a function of modulation amplitudes g_2 with alternating spacing Ω and 3Ω in the frequency dimension. (b1) Intensity distributions of the eigenstates $|\Phi_{a(b),n}|^2$ for the localized edge states at B (blue) labeled in Fig. S1(a) with parameters $g_2 = 0.6\Omega$ and $\Delta\varepsilon = 0.0823\Omega$. (c1) Intensity distributions of the eigenstates $|\Phi_{a(b),n}|^2$ for the localized edge states at C (pink) labeled in Fig. S1(a) with parameters $g_2 = 0.9\Omega$ and $\Delta\varepsilon = 0.8053\Omega$. (b2) and (c2) Corresponding simulation results of intensity distributions $|s^{\mathrm{out}}_{a(b),n}|^2$ for the output field with left boundary excitation at the time $t = 10^4\Omega^{-1}$. The red arrow indicate the frequency supermode B_{-6} in the left boundary is excited. Other parameters are $g_1 = 0.1\Omega$, $\phi_1 = \phi_2 = 0$, and $\gamma = 0.01\Omega$.

*Corresponding authors: ljwang@sjtu.edu.cn, yuanluqi@sjtu.edu.cn

due to the coupling between the quasienergy drive frequency and the frequency dimension of the Floquet SSH lattice, the smallest possible temporal period of the resulting Hamiltonian has been changed as π/Ω. It is critical to specify this value, otherwise, it triggers additional folding of the bands on the energy axis, which obscures the band gap. Figure S1(a) shows the quasienergy bandstructure of the synthetic frequency Floquet SSH model with the spacing between supermodes 1:3, which has the similar bandstructure but different smallest possible temporal period as the one with the spacing between supermodes 1:2, i.e., Fig. 2(b) in the main text. It is worth noting that at this time, the obvious breaking of the degenerate edge states at zero mode can be seen at the larger g_2. As shown in Figs. S1(b1) and S1(c1), the intensity distribution also exhibits the localization on the left boundary of the synthetic lattice at zero mode [labelled by B in Fig. S1(a)] and π-mode [labelled by C in Fig. S1(a)]. Simulations in Figs. S1(b2) and S1(c2) verify the localization effect, and the total intensity for the output field at the zero mode is still larger than π-mode.

II. FLOQUET SSH LATTICE MODEL IN THE REAL SPACE

In this section, we prove that the break of the degenerate edge states and the generate of edge states at the 0 and π energy bandgaps are unique features of the SSH model in the synthetic frequency dimension. For this purpose, we start with the Hamiltonian

$$H = \omega_a \sum_n a_n a_n^\dagger + \omega_b \sum_n b_n b_n^\dagger + \sum_n [2g_1 \cos(\Omega t + \phi_1) a_n^\dagger b_n + 2g_2 \cos(\Omega t - \phi_2) a_{n-1} b_n^\dagger + \text{h.c.})], \tag{S3}$$

which describes a Floquet SSH lattice model in the real space consisting of photonic resonators of resonant frequencies ω_a and ω_b, respectively. The modulation amplitudes in each cell is $2g_1$, between adjacent cells is $2g_2$, the modulation frequency is $\Omega = \omega_a - \omega_b$, and the modulation phases are ϕ_1 and ϕ_2. The Floquet spectrum of the system can be obtained by using the same Floquet analysis method in the main text.

In Supplementary Fig. S2, we plot Floquet quasienergy bandstructures of the Floquet SSH lattice model in the real space. For the weak coupling regime $g_1 = 0.01\Omega$, the quasienergy bandstructure is in agreement with the result of $\tilde{H}_{\text{RWA}}$ in the main text Fig. 2(a1), which has a pair of edge states at zero mode. Figs. S2(b) and S2(c) show that with increasing modulation strength, the RWA becomes invalid and the bulk state folds down several times in a single temporal Brillouin zone. However, the system here only has a single modulation frequency Ω, which will not couple to any other frequencies, thus edge states with energy splitting will not appear.

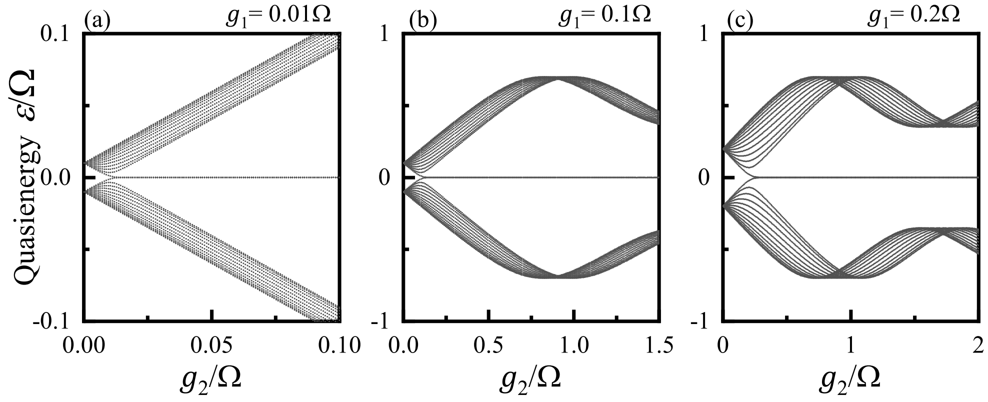

FIG. S2: (color online) Floquet quasienergy bandstructures of the Floquet SSH lattice model in the real space as a function of modulation amplitudes g_2 with (a) $g_1 = 0.01\Omega$, (b) $g_1 = 0.1\Omega$ and (c) $g_1 = 0.2\Omega$. Other parameters are $\phi_1 = \phi_2 = 0$.

III. LONG-RANGE COUPLING FLOQUET SYNTHETIC SSH LATTICE

An important advantage of synthetic frequency dimension systems is that long-range coupling can be easily achieved by choosing modulation formats. Therefore, the influence of the long-range coupling on the topological properties of this particular Floquet synthetic SSH model can be conveniently studied. Here we consider additional modulation terms with a modulation frequency 3Ω, which offer the Next-Nearest-Neighbor (NNN) hopping along the frequency

axis. Thus the corresponding Hamiltonian is

$$
\begin{aligned}
H = & \sum_n (\omega_n + \frac{\Omega}{2})\ a_n a_n^\dagger + \sum_n (\omega_n - \frac{\Omega}{2}) b_n b_n^\dagger + \sum_n J(t)(a_n^\dagger b_n + a_{n-1} b_n^\dagger + \text{h.c.}) \\
& + 2\kappa_1 \cos(3\Omega t + \phi_3)(a_n^\dagger a_{n+1} + \text{h.c.}) + 2\kappa_2 \cos(3\Omega t + \phi_4)(b_n^\dagger b_{n+1} + \text{h.c.}),
\end{aligned} \tag{S4}
$$

where $2\kappa_1$, $2\kappa_2$ are the additional modulation amplitudes and ϕ_3, ϕ_4 are the additional modulation phases.

By employing the Floquet analysis, we show the corresponding energy bandstructure of the extended Floquet synthetic SSH model with NNN hopping in Fig. S3. The comparison between Fig. S3(a) and S3(b) indicates that, under RWA, edge states at zero modes gradually approaches 0 as the coupling strength g_2 increases, but which is lift for the case in the Floquet analysis. One can see that it is not necessary to increase g_1 and g_2 in the same proportion. When g_1 is small and only g_2 is large, the system already exhibits the Floquet topological features with breaking of the degeneracy of the edge state at zero modes. Once g_1 is further increased to 0.1Ω as shown in Fig. S3(c), a series of edge states caused by band folding at the 0 and π energy bandgaps still appear. Therefore, exotic Floquet topological phenomena in synthetic frequency dimensions still exist under long-range coupling, and the bandstructure gets further modified compared with that in a conventional SSH lattice.

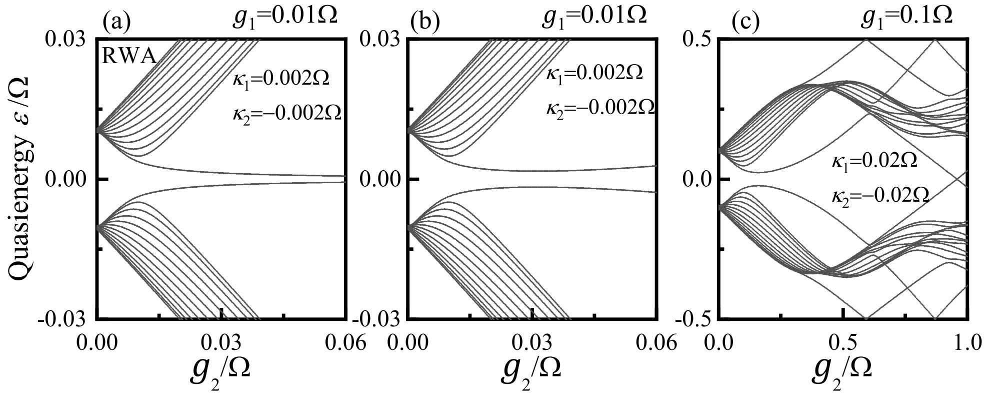

FIG. S3: (color online) Floquet bandstructure of the generalized synthetic frequency SSH model with next-nearest-neighbor(NNN) hopping term as a function of modulation amplitudes g_2 (a) with RWA $g_1 = 0.01\Omega$, without RWA (b) $g_1 = 0.01\Omega$ and (c) $g_1 = 0.1\Omega$. Other parameters are $\phi_1 = \phi_2 = \phi_3 = \phi_4 = 0$.
